# Freshwater systems and ecosystem services: Challenges and chances for cross-fertilization of disciplines

**DOI:** 10.1007/s13280-021-01556-4

**Published:** 2021-05-13

**Authors:** Ágnes Vári, Simone A. Podschun, Tibor Erős, Thomas Hein, Beáta Pataki, Ioan-Cristian Iojă, Cristian Mihai Adamescu, Almut Gerhardt, Tamás Gruber, Anita Dedić, Miloš Ćirić, Bojan Gavrilović, András Báldi

**Affiliations:** 1 Lendület Ecosystem Services Research Group, Centre for Ecological Research, 2-4 Alkotmány utca, 2163 Vácrátót, Hungary; 2 Department Ecohydrology, Leibniz-Institute of Freshwater Ecology and Inland Fisheries (IGB), Justus-von-Liebig-Str. 7, 12489 Berlin, Germany; 3 ELKH Balaton Limnological Research Institute, Klebelsberg K. u. 3, 8237 Tihany, Hungary; 4 Institute of Hydrobiology and Aquatic Ecosystem Management, University of Natural Resources and Life Sciences, Vienna, Gregor-Mendel-Straße 33, 1180 Vienna, Austria; 5 WasserCluster Lunz - Biologische Station, Dr. Carl-Kupelwieser-Prom. 5, 3293 Lunz/See, Austria; 6 Department of Civil Engineering, Faculty of Engineering, University of Debrecen, Ótemető u. 2-4, 4028 Debrecen, Hungary; 7 Center for Environmental Research and Impact Studies, University of Bucharest, Bulevardul Nicolae Bălcescu nr. 1, Bucureşti, 030167 Romania; 8 Research Center for Systems Ecology and Sustainability, University of Bucharest, 050095 Bucharest, Romania; 9 Limco International GmbH, Wollmatinger Str. 22, 78467 Constance, Germany; 10 WWF Hungary, Álmos vezér útja 69/A, 1141 Budapest, Hungary; 11 Biology Department, Faculty of Science and Education, University of Mostar, Rodoč bb, 88 000 Mostar, Bosnia and Herzegovina; 12 Institute of Chemistry, Technology and Metallurgy, University of Belgrade, Njegoševa 12, 11000 Belgrade, Serbia; 13 Department of Physical Geography, Geographical Institute “Jovan Cvijić”, Serbian Academy of Sciences and Arts, Djure Jakšića 9, Belgrade, Serbia

**Keywords:** Aquatic ecosystems, Blue infrastructure, Ecosystem functions, EU Water Framework Directive, Inland waters

## Abstract

Freshwater ecosystems are among the most threatened in the world, while providing numerous essential ecosystem services (ES) to humans. Despite their importance, research on freshwater ecosystem services is limited. Here, we examine how freshwater studies could help to advance ES research and vice versa. We summarize major knowledge gaps and suggest solutions focusing on science and policy in Europe. We found several features that are unique to freshwater ecosystems, but often disregarded in ES assessments. Insufficient transfer of knowledge towards stakeholders is also problematic. Knowledge transfer and implementation seems to be less effective towards South-east Europe. Focusing on the strengths of freshwater research regarding connectivity, across borders, involving multiple actors can help to improve ES research towards a more dynamic, landscape-level approach, which we believe can boost the implementation of the ES concept in freshwater policies. Bridging these gaps can contribute to achieve the ambitious targets of the EU’s Green Deal.

## Introduction

Nature is valued by people in many different ways, while at the same time natural ecosystems are being degraded and destroyed at an unprecedented scale (Díaz et al. 2015; European Environment Agency (EEA) 2019). One approach to assess and convey the value of nature to mankind relies on formulating the vital dependence of humans on nature in terms of ‘ecosystem services’, or as ‘nature’s contribution to people’ (Díaz et al. 2015; Pascual et al. 2017). In order to enhance policy uptake and the chances of success of conservation and restoration attempts, high-level science-policy platforms have been established that served policy makers with integrated and agreed information on the extent of biodiversity and ecosystem loss and also presented projections to the future (Díaz et al. 2015; IPBES 2018a; European Environment Agency (EEA) 2019).

Freshwater ecosystems are among the most threatened in the world, with global declines in their area by 64% from 1997 to 2011, and for Europe by 50% from 1970 to 2008 (Costanza et al. 2014; IPBES 2018a; Gozlan et al. 2019). They are also especially vulnerable to multistressor effects (Borgwardt et al. 2019). Freshwaters—lakes, rivers, wetlands, including floodplains—have always played a major role in the history of humankind and the goods and services they provide are of key importance to our survival and well-being (Wantzen et al. 2016). Systematic reviews list between 20 and 32 ecosystem services (ES), the most frequently mentioned ones being recreation and tourism, water supply, water quality control, habitat provision, erosion prevention as well as food supply and climate regulation (Hanna et al. 2018; Kaval 2019). Freshwater ES studies name numerous provisioning services, like supplying fertile soils for agriculture and places for orchards in the floodplains, reed for construction, drinking water, and food (fish, crustaceans, molluscs) (Reynaud and Lanzanova 2017; Tomscha et al. 2017; Hanna et al. 2018; Hossu et al. 2019). Freshwater ecosystems also provide several regulating services, like groundwater recharge, flood regulation, microclimate regulation, carbon sequestration, water quality control (Bullock and Acreman 2003; Aldous et al. 2011; Tomscha et al. 2017; Hossu et al. 2019) as well as cultural services, such as the existence of spiritual places, their symbolic and aesthetic value, inspiration, giving a sense of place to people and several recreational aspects—swimming, angling, boating (Wantzen et al. 2016; Hanna et al. 2018; Hossu et al. 2019; Thiele et al. 2020). In addition, services like providing habitat for fish, amphibian and bird populations, including spawning grounds and migration as well as seed dispersal (Aldous et al. 2011; Hettiarachchi et al. 2015; Tomscha et al. 2017; Hanna et al. 2018) support the overall functioning of the ecosystem. Hence, it is not surprising that river and lake ecosystems as well as wetlands have the highest estimated per ha value of ES supply of all inland ecosystems (12,512 × 10^12^ $/year and 25,681 × 10^12^ $/year for lakes/rivers and for freshwater wetlands, compared to 3137–4166 × 10^12^ $/year for temperate forests and grasslands) while being the smallest in terms of surface area (0.39% and 0.12% for lakes/rivers and for freshwater wetlands—Costanza et al. 2014).

Despite their importance, research on freshwater ecosystem services (FES) is limited. For example, reviews on riverine ES found only 69–89 studies across the past years (Hanna et al. 2018; Kaval 2019), and 1026 studies for lake and wetland ES together (Xu et al. 2018), while Reynaud and Lanzanova (2017) found 133 studies focusing solely on lake economic valuation. A systematic review on the assessment and conservation management in large floodplain rivers revealed that only 1.6% of the studies addressed ES (Erős et al. 2019), even though considering ES can be highly relevant when assessing the effects of river restoration as shown in the recent study by Funk et al. (2020). On the other hand, a review of ~ 3.000 publications showed that many papers on ES were published in general environmental journals, or specific sectoral journals (forestry, agriculture, etc.), but hardly any in water-related journals (McDonough et al. 2017). It is only in recent years that more comprehensive water-related projects on ES can be found, such as AQUACROSS (Anzaldua et al. 2018; Langhans et al. 2019) and RESI (Podschun et al. 2018).

On the one hand, several reviews (e.g. Martin-Ortega et al. 2015; Tomscha et al. 2017; Hanna et al. 2018; Kaval 2019) identified research gaps in freshwater ES related to the assessment of multiple ES, ES interactions (trade-offs and synergies) and transdisciplinary approaches, which are more of a general nature and not restricted to ES applications in freshwater ecosystems. On the other hand, ‘traditional’ freshwater ecological literature has dealt with a diversity of freshwater-specific issues and developed a set of ecosystem-specific concepts e.g. River Continuum Concept (Vannote et al. 1980), Flood Pulse Concept (Junk et al. 1989), Stable States theory for shallow lakes (Scheffer 1990). Integrating freshwater-related ecological concepts and discussing elementary features of lentic and lotic waters can help advance ES research as well as aquatic management practices.

In this paper we summarise the output of a workshop aimed at identifying knowledge gaps in freshwater ecosystem services (FES)-related research and addressed the following research questions:

What are the challenges and knowledge gaps in freshwater ES studies that are of outstanding importance:

- specifically for the analysis of freshwater ecosystems and their services?
- for the implementation of the ES concept in management and integrated valuation of freshwaters and related policies?
- for future work in ES research in general, where freshwater research can advance ES research?

## Methods

The workshop ‘Aquatic ecosystem services—assessment, management and socio-economic challenges’ took place between 27th and 28th of November 2019, in Budapest, Hungary (http://aquaticES.ecolres.hu/). The workshop aimed to give an overview on the state-of-the-art knowledge on aquatic ecosystem services, from (anthropogenic) pressures to the condition of rivers and lakes and the diversity of benefits that humans obtain from these ecosystems, including the possibilities and potential drawbacks of quantifying natural systems.

The 22 participants were all experts working in the field of freshwater research and/or ecosystem service research and coming from Central and Eastern Europe (from Serbia, Bosnia and Herzegovina, Croatia, Romania, Hungary, Austria, Germany). The workshop comprised three steps (1) introducing participants and some invited presentations as food for thought (2) a world café with two rounds and two groups in each (3) a joint reflection and summary of results.

In the first round of the world café issues were collected that the participants found relevant in their own (freshwater related) experience regarding the application of the ES framework. Lead by the moderators, positive as well as negative experiences were gathered, aspects where the ES framework was found useful and where difficulties were encountered in its application to freshwater ecosystems. In the second round of the world café the participants changed groups. After the moderators wrapped up the first round, the presented difficulties were further developed towards the identification of knowledge gaps. The second day, this collection was structured into emerging clusters, discussed and refined in a joint reflection by the thirteen authors of this paper.

After the workshop, the topics were complemented with an extensive literature review. Thus, while all workshop members framed the study and contributed evidence and ideas, the decisions on the final content were made by the authors of the paper (including decisions on knowledge gap categorization and direction of knowledge transfer). Literature was screened based on keyword searches related to the emerging issues, background knowledge and expertise of the authors and snowballing.

Altogether, we identified six major topics, with a number of challenges and knowledge gaps (Table 1). We classified four different types of knowledge gaps: some topics involve real gaps in knowledge which can be called “conceptual or relationship knowledge gaps”, others reflect gaps in methodological implementation (“methodology gaps”). In some cases, knowledge is theoretically available, but not sufficiently widespread (see also 3.6): transfer is limited either geographically (e.g. from Western Europe towards South-east Europe) or between sectors or organizations (e.g. from academia towards management) or simply not well enough known (possibly also because methodology is not easy to implement)—we can refer to these rather as “challenges” that need attention and fostering.

**Table 1:** Overview of challenges and knowledge gaps summarizing special features related to freshwater ecosystems and their assessment within an ecosystem services (ES) framework with good examples (possible steps) towards a solution

No.	Special features	Possible steps	KT	Good examples	KG
1	*Unique features of freshwater habitats and their role in the supply of ESs*				
	Unique spatial structure—more complex to model	Combine 3D and linear features of waters (embedded in terrestrial ecosystems) in holistic watershed models	F→ES	Nedkov & Burkhard (2012) show how results from hydrological modelling (KINEROS) in the municipality of Etropole, Bulgaria, were extracted and recombine based on ratios of landcover in a matrix-type approach. This is a good example for integrating flood regulation in the watershed, but less successful for floodplain processes. Calculating ES “backwards”, from a specific model to a matrix might be also feasible for other areas of ES assessment.	2
	Directional connections, flow hierarchy, connectivity—fragmentation effects – more complex to manage	Link ES assessment to basic ecological/hydrological concepts of riverine systems	F→ES		2 & 3
	Strong connections: longitudinal & lateral (flood pulses, water level fluctuations) & subsurface (invisible connections with groundwater)	Add sub-surface waters to models	F→ES	Some databases available, e.g. EU-SoilHydroGrids, but linkage to models difficult. https://esdac.jrc.ec.europa.eu/content/3d-soil-hydraulic-database-europe-1-km-and-250-m-resolution	2
	Mapping small/linear extent	Harmonise coarse-resolution terrestrial maps and fine-scale maps of small freshwater bodies		We are not aware of any good examples.	2 & 3
2	*Find solutions across ecological and administrative scales for ES assessments*				
	Administrative borders limiting watershed approach	Co-operations: integration of ES assessments across administrative units (aligned to basin boundaries)	F→ES	More funding for transboundary assessments; some pilot projects available, e.g. Interreg IDES, which aims at improving water quality in the Danube river and its tributaries by integrative floodplain management based on Ecosystem Services, by joining 10 countries along the Danube. http://www.interreg-danube.eu/approved-projects/ides	4
	Upstream–downstream and lateral flooding issues mirrored in social & management problems	Upstream—downstream governance as example for good management practices	F→ES	A good example is provided by the ‘Upstream Thinking’ initiative of the regional water company in Cornwall (UK): they work with farmers to improve the quantity and quality of water through land use change as an alternative to engineering and chemical treatment options, emphasizing their responsibility regarding spatially remote effects of their actions (Schaafsma et al. 2015).	4
	Diversity of dataset scales & resolution	Integrate data across institutions and countries	F↔ES	A first step is the common collection of data, e.g. WISE WFD data. However, integration needs to be solved as a next step. www.eea.europa.eu/data-and-maps/data/wise-wfd-4	2 & 4
	Remote effects scantily quantified	Understand the distance functions of spill-over/zonal effects of water bodies and wetland areas	F↔ES	Some knowledge on applications of groundwater recharge and its remote effects, e.g. by successfully creating numerous water holes in India in order to revitalize surrounding land (Everard 2015) but very few sufficiently exact and generalizable quantifications.	3
3	*Integrated valuation of freshwater ecosystem services*				
	Monetization perceived as dangerous	Integrate and emphasize non-monetary values in assessments	ES→F	Ranking preferences e.g. for differing management options under consideration for wetland restoration planning in Rhode Island, USA makes non-monetary values integrateable into the decision-making process (e.g. Martin & Mazzotta, 2018).	2 & 4
	Values dependent on socio-ecological system setting	Streamline scenario analyses for different socio-ecological settings	ES→F	Estimation of service flow in biophysical units per area and year in Nordic catchments and then estimation of a monetary value for each service in each scenario (Vermaat et al. 2020). They posit that the estimation of total economic value would work as a tangible indicator for comparative use in scenario evaluations and in communication with policy makers.	2 & 4
	ES indicators & assessments diverse and difficult to standardize	Provide unified and comparable indicators and valuation systems with intercalibration techniques	F↔ES	Good example is the River Ecosystem Services Index (RESI) developed for German rivers and calibrated at several sites, incorporating also WFD-used features (Podschun et al. 2018).	2 & 3
	Multiple aspects to reconcile (social, conservation, etc.)	Promote decision-support and other frameworks for landscape-level decisions (based on multifunctionality and conservation focus)	F↔ES	Multifaceted problem-solving and decision making is developed by Colloff et al. (2019), including new ways to connect with the societal decision system. Applying scenario analysis, the results can be used to visualize trade-offs that affect livelihoods, human wellbeing, water resources & the environment.	2 & 3
4	*Enhancing databases and methods*				
	Multitude of different databases in data-developed regions	Develop methods to integrate different databases across disciplines and across countries, with intelligent databases	ES→F	We are not aware of any good examples.	2
	Lack of data in data-poor regions – less complex assessments possible	Assess freshwater ES on large scale in data poor regions, develop ‘quick & dirty‘methods, test downscaling	ES→F	Enhance funding for basic/monitoring data collection, especially in South-east Europe. While there are some rough estimating methods available for terrestrial ES (e.g. crop provisioning), for water related they are much more complex (see Vallecillo et al. 2019 for both).	1 & 2
	Accuracy and uncertainty of assessments often not visible or not assessed	Visualize uncertainty levels (e.g. flag data/results), compare with higher tier models, test upscaling	F↔ES	A useful approach towards assessing uncertainty is presented in the EU Ecosystem assessment (Maes et al. 2020). With the use of basic uncertainty categories derived from the used methodology and quality of datasources gives a first approach of how to categorize uncertainty when otherwise not quantifiable (i.e. no model calculations possible).	2
5	*Incorporate ESs into management and increase EU policy coherence on water*-*related ESs*				
	EU policy lacks coherence on water-related ES	Develop & promote guidance on integration between ES assessments, policy, and specific measure	F↔ES	Several overview studies compare different policies, e.g. Bouwma et al. (2018) analysing 12 policies, which show some coherence, but further integration based on ES is needed.	2 & 4
	Need to recognize rivers and lakes as blue infrastructure	Recognize rivers and lakes as blue infrastructure, link freshwater ES to up-to-date policy directions	F↔ES	Within the project ‘ENABLE’ a framework was developed and applied in six pilot cities, that evaluates functionally connected green and blue features (Andersson et al. 2019) with three key factors including : 1) the interactions among green, blue, and built infrastructures, 2) institutions 3) peoples perceptions and values. The framework can be used to support more effective urban planning, decision-making and implementation of green & blue infrastructure. The full extent of its potential is not yet established in research & policy, further efforts are needed. https://www.iucn.org/regions/europe/our-work/nature-based-solutions/enable-improving-green-and-blue-infrastructure-cities	4
6	*Improve communication, education and knowledge transfer*				
	Gap in knowledge transfer towards policy makers, conservation practitioners and from high GDP to lower GDP countries	Improve communication efficiency towards decision makers and practitioners; involve knowledge brokers	ES→F	With constant negotiation between researchers and knowledge users (policy actors), knowledge brokering could provide Finnish civil servants with pre-digested and fit-for-purpose information about the ES indicators and thus help urban green space planning (Saarela & Rinne 2016).	4
	Positive psychological effects	Use emotional attachment to enhance communication	F↔ES	“Big Jump for Europe’s Rivers” calls for greater protection for continent’s rivers and lakes—people participate in simultaneous events in 18 European countries every year as part of the Big Jump—jumping, diving, wading, kayaking and swimming in streams and ponds, rivers and lakes. WWF & locals jointly organised > 160 events.	4
	Dynamic/periodic changes in freshwater ecosystems are difficult to manage with static approaches	Integrate lessons from traditional ecological knowledge on the coexistence of people and dynamic aquatic habitats	F↔ES	We are not aware of any good examples.	4

Knowledge transfer: we indicated where freshwater research can advance ES research (F→ES), where ES research can assist FES research (ES→F) and where knowledge development in both is needed (F↔ES). KG: Knowledge gap types: 1—data gap (raw data not available, e.g. status of some water bodies); 2—methodology gap (methodology is not (readily) available, e.g. to combine datasets from different sources or scales); 3—conceptual or relationship knowledge gap (knowledge is not available); 4—transfer gap (knowledge available, but not to all relevant participants; e.g. geographic inequality, or between research and implementation/management)

We also evaluated the specific findings from the point of view of knowledge transfer: wherever knowledge or methods of assessment/management are more developed, better accepted or work in some way better regarding freshwater ecosystems than ES research in general, we mark this, as well as the other way round: issues/practices that work better in more general ES approaches and are less successfully implemented in FES studies.

In the following sections, we present the emerging issues and complement them with suggestions on how to address these complex questions.

## Results

**Fig. 1:**
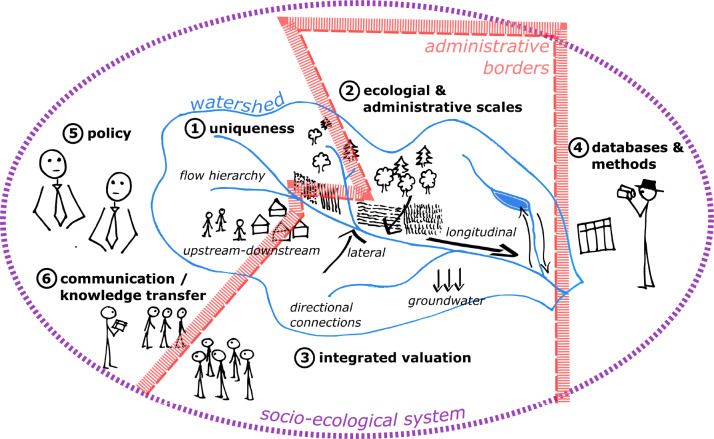
The unique features of freshwater ecosystems (1) are at the very core of all of the discussed issues. These are nested within ecological and administrative borders (2, blue-watershed, red-administrative border), that makes integrated valuation of the ES necessary (3), to which issues regarding datasets and methods are related (4). Accessibility, coverage and availability of both, data and methods, are shaped by the features of the socio-ecological system (violet) defined by management and policy (5) as well as knowledge exchange among stakeholders (including policy actors and management) (6). For details regarding the six specific issues, see Table 1

We developed a conceptual framework (see also Fig. 1): at the core of most issues identified are several features which are unique to freshwater ecosystems and have a firm (bio)physical basis. These are embedded in a landscape that is divided into ecological and administrative units. Both,’unchangeable’ biophysical features as well as relatively fixed ecological entities need to be reconciled with man-made administrative units. Integration between the different relevant sectors—as well as between different valuation approaches resulting in an integrated valuation of ES—might be one way forward. Integrated valuation of ES itself holds a number of challenges regarding datasets and methods like accessibility, geographical coverage and availability for example. These challenges are shaped by properties of the socio-ecological system (research infrastructure, funding, etc.), which in turn can be influenced by policy. Both of these are human-made and can be changed relatively easily, at least compared to biophysical attributes. The exchange of knowledge (between science and other stakeholders, like policy actors and managers) and the enhancement of knowledge exchange—factors that rely on all of us—is the key to ensure the preservation the functioning of freshwater ecosystems.

### The unique features of freshwater ecosystems and their role in the supply of ES

One of the most prominent features of freshwaters is their unique spatial structure that distinguishes them from terrestrial habitats, influencing the spatial and temporal distribution of ES and their interactions. Waterbodies are embedded within the terrestrial landscape constituting’transitional systems in space and time’ (Hettiarachchi et al. 2015). As the watershed area is much greater than the surface area of either rivers or lakes, interactions between land and water are more pronounced when we include the strong impact of land on water (e.g. through fertilizer input) emphasizing the critical role of connectivity and interfaces for the overall functioning of freshwater systems. The strong directionality of the flow of material and energy distinguishes lotic freshwaters from terrestrial systems, while fluctuations in water level are crucial for wetlands and lakes. Both constitute changes in extent and shoreline and have the potential to affect biota as well as stakeholders. In riverine habitats, interactions resulting from the distinct directionality and unique connectedness of rivers across broad spatial scales strongly influence local-scale habitat features and organization of the biota (Thorp et al. 2013; Erős and Grant 2015), with inevitable effects on ecosystem functions and services.

Due to their linear structure, rivers are especially susceptible to fragmentation effects, like those resulting from building hydroelectric power plants (transversal) or by building levees along river banks (longitudinal). In fact, these connectivity relationships may be the most fundamental difference between riverscapes and terrestrial landscapes because the linear structure of rivers allows for disproportionately large effects of barriers. Studies proved that hydropower dams can cause enormous degradation of biodiversity and ecosystem services by impeding connectivity in freshwater networks (Wu et al. 2003; Poff et al. 2007; Borgwardt et al. 2019). In terrestrial habitats, one single obstacle could rarely cause such disproportionate harm, as circumventing barriers is more feasible (Erős and Lowe 2019). Thus, whereas terrestrial ecosystems are often valued as more or less closed entities, with local-scale supply of ES, this is not possible for water-related ecosystems and services, as inputs from other ecosystems and catchment-level effects have to be taken into account (Bennett et al. 2009; Thorp et al. 2010; Qiu and Turner 2013; Hanna et al. 2018). Resulting from the high connectedness of aquatic ecosystems, a quantification of interactions between their ES is challenging (c.f. “Integrated valuation of freshwater ecosystem services” section) but all the more important due to the potential impact of management measures on both terrestrial and freshwater systems (c.f. “Incorporating ES into management and to increase EU policy coherence on water related ES” section).

Waters are not only connected on the surface, but also in an invisible way, to groundwater. Surface water bodies can be connected along aquifers, whereas within the whole watershed, sub-surface and surface run-off connects both terrestrial influences to waterbodies and groundwater. Therefore, ES of surface waters should be managed with regard to hydrologic processes connecting both (Qiu and Turner 2013). Although mainly driven by abiotic factors, groundwater ecosystems can provide numerous ES, which is rarely taken into consideration (Griebler and Avramov 2015; Pinke et al. 2020). Groundwater levels have been declining due to direct water abstraction (pumping) for drinking water and irrigation (Gozlan et al. 2019) for example, but also due to reduced opportunities for recharge. Recharge can occur in wetlands of floodplains, but river regulations in the past centuries resulted in a reduction of potential recharge areas (Bullock and Acreman 2003; Aldous et al. 2011).

Temporal aspects also need to be considered, as due to water level fluctuations the borders of freshwater systems are dynamically changing. The periodic change in size/volume is thus another unique feature of most freshwater ecosystems: regular flood events, potentially occurring both in rivers and lakes, provide an even stronger linkage between terrestrial and aquatic habitats, enhancing lateral connectivity—the connections between the main river/water body and its surrounding floodplains and oxbows. However, droughts and drying also change the boundaries and have massive impacts on ecosystem functioning and ES (Moomaw et al. 2018; Keller et al. 2020). Riparian zones constitute transitional areas between land and freshwater that are of special value for biodiversity and ecosystem functioning (e.g. Flood Pulse Concept, Junk et al. 1989; Wantzen et al. 2016; Tomscha et al. 2017). However, management of an ecosystem that regularly changes its extent poses special challenges, especially if the pulsing is to be reconciled with human needs. Haines-Young and Potschin (2010) classified ES based on their spatial characteristics, and listed several basic water related ES ‘water regulation/flood protection’, ‘water supply’, ‘sediment regulation/erosion control’, ‘nutrient regulation’ as ‘directional flow related’, in contrast to local scale or global, but non-directional ES. Nevertheless, the majority of later ES mapping and assessment works neglected the more complex spatial aspects and concentrated on the easy-to-map local or ‘proximal’ ES. Concepts of ‘service providing units’ and ‘service benefitting areas‘have been developed, but are still challenging to implement (Syrbe and Walz 2012). Therefore, frameworks attempting to adapt general ES approaches to waters, and rivers need to take directional flow into account, e.g. by integrating hydrologic models into their frameworks (e.g. Keeler et al. 2012; Hallouin et al. 2018).

Mapping habitats, land cover/land use, or ecosystems constitutes an essential task for ES assessments. The narrow, linear shape of streams and the small size of many lentic waters is a challenge for proper representation in the maps: if the grid used is too coarse, the extent of the ecosystem might be strongly underrepresented or completely missing from the maps (e.g. Tomscha et al. 2017). Also, the correct mapping/representation of the terrain elevation is crucial in order to be able to model water flow directions properly.

### Finding solutions across ecological and administrative scales for ES assessments

Spatial scaling is an issue that has been around for decades with some more recent advances based on fine-resolution remotely sensed data (Tomscha et al. 2017). Deciding about the right scale—or multiple scales—for an ES assessment is of great importance, as different scales can yield different results (Friberg et al. 2017; Hanna et al. 2018). Most water-focused studies use a watershed approach, and this is also suggested as the appropriate scale for management by the EEA (Hanna et al. 2018; European Environment Agency 2019), as well as for Water Framework Directive assessments (EC 2003). Nevertheless, there are still a great number of studies complying with jurisdictional boundaries, as this is the scale for administrative actions, including national funding and regulations (Mihók et al. 2017). This approach however, cannot give optimal results from an ecological point of view (Kaval 2019). International and/or cross-border co-operation could help in tackling this problem.

Directional flow also entails a line of social and management issues, where the effects of upstream decisions are being carried on by people and ecosystems further downstream, potentially to different administrative units (Thorp et al. 2006; Brauman et al. 2007; Hanna et al. 2018). Sensibilization towards this fact has been successfully applied in the UK for example (‘upstream thinking’—Schaafsma et al. 2015).

Questions regarding how integration between different scales should be implemented can also arise at the data level, when working with a diversity of datasets. Datasets from different sources, with different spatial scales, resolution and units need to be transformed and integrated into one comprehensive dataset for large scale studies. As databases—even within countries (e.g. Engloner et al. 2019)—are developed by different agencies or institutions, their integration poses difficulties and is often missing (for example regarding hydrological and meteorological data).

The spill-over (zonal/remote) effects of water bodies—effects of water that are detectable across a wider area within their surroundings—are not sufficiently known, e.g. at what distance water bodies can have an effect on microclimate via evaporation, potentially providing climate regulation even at regional scale, or how retaining water in floodplains effects groundwater levels in the surrounding areas in the long run (Bullock and Acreman 2003; Pinke et al. 2020). Changes in local and regional air temperature could be detected and analysed by remote sensing, backed with data provided by local in situ sensors for calibration.

### Integrated valuation of freshwater ecosystem services

Assessment of ES is often seen as synonymous with monetary valuation since at least Costanza’s work on the world’s ecosystem services in (1997). Putting monetary values on ‘nature’ is a critical issue that crystallized during the workshop, as monetization is perceived as dangerous, which was the most controversial experience that participants reflected on. This shows that the misconception that monetary valuation is the only way to make ES comparable is still persistent outside the ES community and the fact that the ES concept embraces a much wider range of values should be communicated widely (Schröter et al. 2014).

Focusing on non-monetary values, like the perceived importance of different FES, taking a deliberative approach with inclusion of traditional ecological knowledge, preferences of local stakeholders as well as presenting bio-physical values wherever possible can be a good solution towards a well-balanced assessment, e.g. as multi-criteria decision analysis, elicitation of socio-cultural preferences or by analysing social networks (Martín-López et al. 2012; Gómez-Baggethun et al. 2016; Martin and Mazzotta 2018). The value of ES, no matter if monetary or non-monetary, depends on various factors. Monetary value depends especially strongly on the demand and the examined economic situation, e.g. the availability of the specific asset (Bateman et al. 2011; Reynaud and Lanzanova 2017). Demand for a service however, might change quickly, if the societal setting or the supply changes. The perceived value of ES has been shown to depend on the viewpoint of the stakeholders (Martín-López et al. 2012; Paudyal et al. 2018; Hossu et al. 2019). Changes in both the social and the ecological system—including land use-changes—can therefore lead to very different valuation results, both in monetary and non-monetary terms (e.g. Shackleton et al. 2018). Scenario analyses might shed light on anticipated changes in ES value as well as adopting values from other regions to a hypothetical situation, similar to the benefit transfer technique widely used for economic valuation of ES (e.g. Reynaud and Lanzanova 2017; Decsi et al. 2020; Vermaat et al. 2020).

Assessing single ecosystem services is one step. However, the strength of the ecosystem services concept lies in assessing multiple ES at once for underpinning holistic management measures. For aggregating multiple ES, a common denominator is needed, which can either be achieved by monetization (Reynaud and Lanzanova 2017), but also by other quantitative methods, e.g. hotspot analysis (Qui and Turner 2013; Schulp et al. 2014; Tomscha et al. 2017). Relative scales—e.g. an ordinal scale with scores from 1 to 5, as often used in ES matrix applications—seem to offer an easy solution, but should be handled with care and not be mistaken for interval or ratio values, that can actually be added up (Czúcz et al. 2018). In order to give relative scales a meaningful interpretation, they need to be standardized and connected to biophysical values.

The Water Framework Directive (WFD; Poikane et al. 2014) is a valuable tool for evaluating the ecological quality/potential of freshwater systems on a relative scale, where biological and chemical indicators are combined in an intercalibration process and used to evaluate water body quality and give guidance on the necessary management needs. The WFD monitoring could be complemented by an ES valuation system in the future as there are already several direct and indirect links (Kistenkas and Bouwma 2018). An adaptation to terrestrial ecosystems based on a similar, systematic intercalibration process to assess the ecological quality could open up new directions in the development of a terrestrial ES valuation system. The approach developed in the RESI (River Ecosystem Service Index, Podschun et al. 2018) project allows the integration of the WFD relative scores and combines them with additional datasets (such as land use, digital elevation model, soil maps etc.) towards an ES assessment including up to 15 ES relevant in rivers and floodplains. Thereby, all ES values are based on individual indicators and models that are finally valued on a relative scale from very low (1) to very high (5) service provision (Podschun et al. 2018). This enables an evaluation of freshwater management scenarios based on the change in overall functionality of the ecosystem, as e.g. shown for a 75 km stretch of the Danube in Stammel et al. (2020). As the use of relative scales is already established in the WFD, stakeholders’ acceptance towards relative ES scales might be higher than for monetary approaches.

While there are several frameworks according to which landscape-level decisions could be made (optimization, e.g. according to pareto-optimal combinations of ES—Vallet et al. 2018), within the ES-related topics, it is often multifunctionality that is promoted as the best solution (Sanon et al. 2012; Funk et al. 2020). Sensitive and protected areas might however not always be outstanding in terms of multifunctionality. Along these lines, there is an on-going debate in nature conservation: whether land should be used as multifunctional as possible (‘land-sharing’) (e.g. assessed for floodplains: Funk et al. 2020), or whether there should be designated areas, for one specific or a set of prioritized functions (e.g. for conservation, ‘land sparing‘). These approaches could be combined using spatial optimization, in which win–win solutions are sought by accounting for ES delivery in each scenario (Erős et al. 2018).

### Enhancing databases and methods

With remote sensing and processing and big (EU-wide) monitoring schemes, the availability and also the diversity of datasets has increased, but so has the effort to overview them and find the best/available datasets. This enables EU-wide ES assessments on the one hand (Grizzetti et al. 2019) but also offers an opportunity for downscaling (Aldous et al. 2011) that might be especially valuable in data-scarce regions (e.g. towards SE Europe). The development of intelligent databases that compile themselves based on a pre-defined search algorithm within an (internet-based) application could be an innovative solution (e.g. Ames et al. 2012). The use of social media and citizen-science based data is an emerging field in environmental research that has mainly been used to assess cultural ES but also to monitor aquatic ecosystems (Ghermandi and Sinclair 2019). Despite the increasing availability of data and coordinated attempts to gather more (e.g. as part of the WFD implementation), there are still large information gaps on the status of freshwater ecosystems: according to the EEA (2019), the status of 40% of these ecosystems is still unknown, while outside of the EU, data is mainly focused on protected areas, leaving other areas’ status in the dark.

The usual way of developing ES assessments is based on gathering existing knowledge via consulting experts (IPBES 2018b). However, there are still several areas, especially within freshwater environments, where appropriate evidence is missing or assessed only with limited confidence. Here, often small-scale modelling tools exist, which are not feasible at larger scales (see scaling issues above). Building and testing some ‘quick-and-dirty’ methods to give a rough estimate on ES within a reasonable time frame are needed. Here, the ES matrix approach (Burkhard et al. 2009; Jacobs et al. 2015) that combines ecosystem types with ES via look-up tables has proven to be a valuable tool that still needs to be adapted to local conditions. An assessment of uncertainty is highly recommended, albeit rarely performed (Burkhard et al. 2009; Campagne et al. 2020; Maes et al. 2020). As many aspects within freshwaters are more interconnected (see above), it is probably more difficult to develop easy-to-implement ES assessment methods than it is for terrestrial ecosystems, while for some ES it is simply not possible. For example, nutrient retention still poses a great challenge as very detailed information on relevant processes is required for a thorough quantification (Grizzetti et al. 2019).

Modelling approaches for ES encompass a wide variety of methods and tools (Schulp et al. 2014; Hanna et al. 2018): from the very simple matrix models to somewhat more refined, but still land-use based models, including spatial rules (Kienast et al. 2009; Czúcz et al. 2018; Arany et al. 2019) and up to higher tier models, which are often process-based, empirical or statistical models (Schulp et al. 2014). For assessing ES, highly developed, data-intense modelling tools are mainly available for specific fields and at local to regional scales, e.g. hydrological models (e.g. SWAT, Hydrus1-D). If large scale ES assessments are to be completed or multiple ES are to be assessed at the same time (e.g. national MAES), simpler models are more often the only feasible ones, due to limited resources. With matrix-based modelling it is hardly possible to include any directional influences, which limits applicability when modelling ES related to the flow of water. Comparing simple models with higher tier models offers the opportunity to assess uncertainty. Upscaling higher tier models from the local/regional/watershed scale to larger areas is not evident, but potentially feasible and needs testing (Grêt-Regamey et al. 2015; Hanna et al. 2018).

### Incorporating ES into management and to increase EU policy coherence on water related ES

While institutions and governance are recognized to be of key importance for ES co-production (Pascual et al. 2017; Mastrángelo et al. 2019), regarding planning, design and management, there are still several points that hinder implementation. Already before the rise of the ES concept, the IWRM (Integrated Water Resources Management) approach emphasized the importance to connect environmental issues and human well-being, and partly already implemented stakeholder integration, while also aiming at multidisciplinarity (Blackstock et al. 2015; Maynard et al. 2015; Grizzetti et al. 2016a). Still, added value is seen in including an ES approach in river basin management plans by its potential for trade-off analysis, better linkages towards and recognition of human well-being aspects (Maynard et al. 2015; Grizzetti et al. 2016b; Crossman et al. 2019) or in its combination with cost-effectiveness analyses (Boerema et al. 2018).

A general lack of ES-based integration between the different EU-level policies and management measures can be observed regarding the numerous policies related to water, e.g. the Nitrate Directive, the Flood Directive, the Habitat Directive, the Biodiversity Strategy, the Drinking Water and the Bathing Water Directives as well as others on adaptation to climate change (Council Directives 91/676/EEC, Directive 2007/60/EC, 92/43/EEC, 98/83/EC, 2006/7/EC, EC, 2011, 2013), social cohesion (EU Regulation No 1300/2013) and energy efficiency (Council Directive 2012/27/EU). Often different policies either contradict each other, or are disregarded by one another (Naumann et al. 2011).

Putting for example measures of the WFD and flood directives in direct relation to their potential effect on ES delivery can help to compare consequences of different measures in a systematic way (Schindler et al. 2016; Hornung et al. 2019). Due to the interactions between ES, trade-offs arise with the implementation of different management measures, typically between (agricultural) provisioning and cultural ES (Hornung et al. 2019).

Freshwaters can also be seen as ‘blue infrastructure’ (EC 2013a, b). The importance of green and blue infrastructure is also acknowledged in the EU Biodiversity Strategy for 2030 (2020/380/EC). An integrated consideration of blue-green infrastructure networks in landscape planning and governance can also help to address societal challenges using nature-based solutions (Albert et al. 2019).

### Improve communication, education and knowledge transfer

Forwarding and communicating cutting-edge findings in science towards society, practice/implementation and policy is vital in order to channel the interest of stakeholders and funding to these areas. For this latter, however, a clear communication between science and decision-makers is needed. This seems to be less efficient in eastern Europe as experienced by the workshop participants—a pattern observed generally in knowledge transfer and accessibility between high- vs low-GDP countries (Karlsson et al. 2007; Jeffery 2014; Blicharska et al. 2017). There is a gap between available knowledge in theory, that is accessible in academic studies and knowledge actually implemented and integrated in management (Langhans et al. 2014; Xu et al. 2018; Lindenmayer 2020). Thus, communication and education targeting nature conservation needs to be enhanced. Better knowledge transfer was also seen by workshop participants as a key to implement and make use of the advantages offered by the ecosystem services concept. In cases where practitioners did have experience with the ecosystem services approach, they perceived it as a very useful tool for involving stakeholders’ perspectives and highly suitable for solving conflicts (own experience; Maynard et al. 2015; Grizzetti et al. 2016b).

For more effective communication, ‘knowledge brokers’ (Saarela and Rinne 2016) who work exclusively on the transfer of knowledge from science to practice could be involved. In this regard freshwater science can learn from ES research and even more from social sciences by adopting truly interdisciplinary methods in order to enhance system-, target- and transformation knowledge for integrated planning (Albert et al. 2019).

Rivers and their floodplains are outstanding in the provision of cultural ES (Thiele et al. 2020) as people are highly connected to water historically, culturally and also emotionally (Corral-Verdugo et al. 2015). This attachment represents a good starting point for communication, education and knowledge transfer regarding conservation issues, while the ES concept helps to communicate these issues with a multitude of stakeholders and to balance between different uses/needs.

Knowledge transfer is also needed from traditional knowledge holders towards science and policy (Molnár and Berkes 2018). The effective integration of traditional knowledge (or indigeneous and local knowledge) is a key priority of the IPBES assessments (Díaz et al. 2015; Mastrángelo et al. 2019). Former cultures settling in floodplains dynamically adapted to flood pulses in contrast to todays’ static structures—this knowledge/practices should be taken more into consideration in formulating alternative water management solutions (see also Wantzen et al. 2016). Historically, one option for floodplain management was the use of oxbow lakes in Hungary—fluvial lakes that were periodically connected to the river during high water levels and used for raising fish stocks, while their flooding decreased flood levels at the same time (Biró 2009; Molnár and Berkes 2018). Nowadays, possibilities of re-vitalizing this management system are discussed intensely (Werners et al. 2009; Derts and Koncsos 2012; Guida et al. 2015).

## Conclusions

In this paper, we highlighted that freshwaters comprise numerous unique features (e.g. high lateral and longitudinal connectivity, directional flow, vertical connections to the subsurface), which make their assessment and management more challenging. These aspects also hold true when including them in an ES assessment framework. Many features presented in the previous sections not only pose problems to be solved, but can also present an opportunity with which we might be able to better address more general questions in ES research, and thereby add to the development of the ES framework. As such, we discussed strong spatial interlinkages that are often incorporated in (water-related) modelling tools, but disregarded in terrestrial assessments; the watershed approach, which takes hydrological borders and not administrative borders as the basis of an assessment; and upstream–downstream issues that show the discrepancy between service providing units and service benefitting areas in a pronounced way in river environments that need to be accounted for in terrestrial environments, too—for these, a number of good practice examples are available from riversides (Schaafsma et al. 2015). Due to their special features, it is more evident to adopt a holistic, integrated approach in many freshwater cases. With this, the multifunctionality within an ecological entity or the interlinking changes related to different sectoral policies can also be analysed better (Schindler et al. 2014; Hornung et al. 2019). Addressing issues like connectivity would be a significant improvement for ES assessments in terrestrial systems that might well fit the concepts of green infrastructure. Harmonizing EU policy related to water and integrating ES assessments into relevant policy pieces could assist in developing target specific measures, in governance as well as in research, like for the incoming EU Horizon Europe research and innovation framework. Focusing on the strengths of freshwater research can help to improve the ecosystem services framework towards a more holistic, landscape-level approach, which we believe can boost realization of conservation attempts and achieving EU and global sustainability goals. As the overview of possible solutions showed, the first steps are already on the way giving rise to more intense cooperations across disciplines and countries.
